# Global Burden of Disease: Digestive Disease Alarm in South Caucasus

**DOI:** 10.7759/cureus.49698

**Published:** 2023-11-30

**Authors:** Arturan Ibrahimli, Altay Aliyev, Elgun Samadov

**Affiliations:** 1 Medicine, Liv Bona Dea Hospital, Baku, AZE; 2 Oncology, Liv Bona Dea Hospital, Baku, AZE; 3 Surgery, Ministry of Health of the Republic of Azerbaijan, Baku, AZE

**Keywords:** global burden of disease (gbd), digestive disease, georgia, armenia, azerbaijan, caucasus, south caucasus

## Abstract

Digestive disease-caused death rates are significantly high in the South Caucasus region. The latest Global Burden of Disease (GBD) data are a subject of discussion and should lead to serious steps to be taken. Azerbaijan, Armenia, and Georgia are three countries in the region with similar cultures but different roots. The problem seems to affect every country in the region with slightly different rates. It is crucial to start investigations into the detailed cause and to take serious steps in order to prevent digestive disease-caused deaths in the region. This letter aims to arouse awareness of the problem in the region.

## Editorial

The digestive system contains the largest number of organs in the body including digestive tract organs like the esophagus and intestines and digestive gland organs like the liver and pancreas [1]. Digestive diseases are complicated diseases with high rates of incidence. In addition, digestive diseases are linked with significant outpatient and inpatient healthcare utilization [2]. Recent data from the Global Burden of Disease (GBD) study discloses an important issue in terms of digestive diseases in the South Caucasus region. It needs to be mentioned that in the GBD study, digestive disease cancers are not included in the digestive disease cause death data.

**Figure 1:**
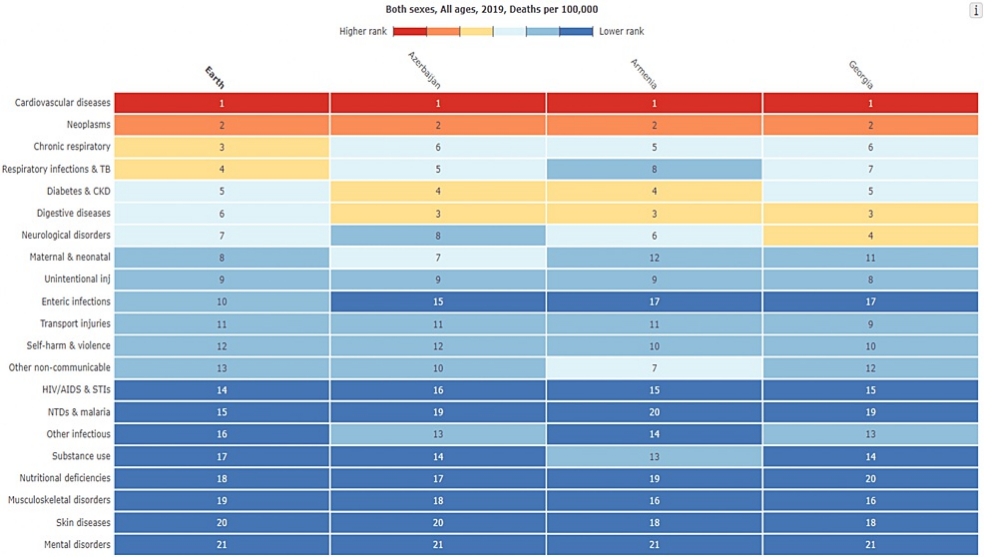
Comparison of the data from GBD 2019. The ranking of the causes of death in globe, Azerbaijan, Armenia, and Georgia. The table is taken from Global Burden of Disease Collaborative Network. Global Burden of Disease Study 2019 (GBD 2019). Seattle, United States: Institute for Health Metrics and Evaluation (IHME), 2020.

Digestive disease-caused death rates are significantly high in the South Caucasus region. Azerbaijan, Armenia, and Georgia are three countries in the region with similar cultures but different roots. The problem seems to affect every country in the region with slightly different rates. The latest data from the GBD obviously show that the death ratio is almost two times greater in the region in contrast with the world average [3]. While the most common cause of death is Cardiovascular cause deaths, Digestive disease is the third cause of death in the region, while it is sixth globally (Figure 1). This letter aims to arouse an awareness of the problem in the region.

While globally, digestive diseases caused only 33.06 deaths per 100,000 population per year, they caused significantly more deaths for the three countries in the South Caucasus [3]. The problem seems to be lighter in Azerbaijan, with 38.38 deaths per 100,000 per year, the burden was more significant in Armenia and Georgia with 56.97 and 58.36 deaths per 100,000 population per year, respectively [3]. It should also be mentioned that since the start of the GBD project (1990), the cause of deaths by digestive diseases has been increasing in the southern Caucasus [3].

The common digestive diseases that caused death were viral and alcoholic cirrhosis, pancreatitis, ileus, peptic ulcer disease, and inflammatory bowel diseases [3]. Cirrhosis and other chronic diseases were found to be the leading cause of death among digestive diseases in the South Caucasus region [3]. Deaths caused by cirrhosis and other chronic diseases in the three countries were found to be almost 50% higher than the global percentage [3]. The worst number was in Azerbaijan, where 4.15% of all deaths were caused by cirrhosis and other chronic diseases, followed by Armenia, 3.98%, and Georgia, 3.16%, while this number is 2.6% globally [3]. The second most common cause was upper gastrointestinal diseases in each country; the worst scenario was in Armenia, where upper gastrointestinal diseases caused 0.8% of all deaths, then Georgia with 0.4% and Azerbaijan 0.29%, the percentage is 0.48% globally showing only Armenia in the region has higher rates than the world average [3]. The third cause of death among digestive diseases differs from country to country. In Azerbaijan, it was “Other Digestive Diseases”; in Georgia, it was paralytic ileus and intestinal obstruction; and in Armenia, vascular intestinal diseases [3]. Deaths caused by inflammatory bowel diseases are relatively low in Azerbaijan compared to Georgia and Armenia [3]. It seems that pancreatitis and abdominal and inguinal hernias are causing challenges in Armenia; in total, they caused 0.25% of all deaths, which is worse than the countries of the same region [3].

But what is the cause of this burden? Each of them has its own etiology, but behavioral status was found to be the most affecting reason for digestive disease-related death in all three countries [3]. The leading behavioral causes were alcohol, smoking, and drug use respectively in Azerbaijan and Georgia, but in Armenia, smoking caused greater damage in terms of digestive diseases than drug use [3]. The other cause was metabolic risks, high body mass index to be precise, which caused 0.14% of all deaths by causing gallbladder and biliary diseases in Armenia [3].

It is possible that the results can be affected by the diagnostic procedures, methods, or screening policies. It would be beneficial for the nations in the region to discuss and share their data on where and how they are better in the exact field to further improve healthcare in the region.

The data comparison is subject to many biases, but it is still a concerning issue that the burden of digestive diseases in the southern Caucasus region is greater than the average of the world. Although the GBD data provides us with a precious asset to start the awareness, the information in it is not very detailed for the southern Caucasus region. In addition, due to the lack of research in the region, it is hard to understand the problem thoroughly.

However, it should be mentioned that the problem is vast and causes a high burden on nations and their economies. The authorities should take the step and investigate the causes by conducting large-scale multinational research to find the root cause of the problem and solutions. For example, the hepatitis C elimination program in Georgia can be a good example, in 2015 they launched the program by taking into consideration not only treatment but also surveillance, education, screening, and linkage to care and they invested in the hepatitis C information system [4]. As a result, Georgia reduced the hepatitis C prevalence by 67% from 2015 to 2021 [5]. Additional steps to eliminate the barriers by integrating screening, care, and treatment services into primary healthcare settings throughout the country are subject to discussion in order to solve the issue in the upcoming years.
